# Supplementary material for: A Systematic Review of the Evidence of Hematopoietic Stem Cell Differentiation to Fibroblasts
Source: Biomedicines. 2022 Nov 28;10(12):3063. doi: 10.3390/biomedicines10123063 (PMC9775738; doi:10.3390/biomedicines10123063)
Supplement: Supplementary file 1 [file biomedicines-10-03063-s001.zip › Supplementary material File S1 - Search strategy.pdf]

PubMed Session Results (28 Sep 2022)

Search	Query	Items found
#4	**#1 AND #2 AND #3**	2,585
#3	**"Cells/etiology"[Mesh] OR "Cell Lineage"[Mesh] OR (("cell"[tiab] OR "cells"[tiab] OR "cellular"[tiab] OR "intercellular"[tiab] OR "peripheral"[tiab] OR "fibroblast*"[tiab] OR "bone marrow"[tiab]) AND ("derive*"[tiab] OR "derivi*"[tiab] OR "origin*"[tiab] OR "source"[tiab] OR "progenitor*"[tiab] OR "provenance"[tiab] OR "root"[tiab] OR "rooted"[tiab] OR "chimer*"[tiab] OR "germ line*"[tiab] OR "lineage*"[tiab]))**	1,688,309
#2	**"Stem Cell Transplantation"[Mesh:NoExp] OR "Hematopoietic Stem Cell Transplantation"[Mesh] OR "Bone Marrow Transplantation"[Mesh] OR (("bone marrow"[tiab] OR "hematopoetic"[tiab] OR "hematopoietic"[tiab]) AND ("transplant*"[tiab] OR "graft*"[tiab] OR "transfus*"[tiab] OR "transfer*"[tiab])) OR "Genetic Heterogeneity"[Mesh] OR "genetic heterogeneit*"[tiab] OR "genetic hetero-geneit*"[tiab] OR "fibroblast heterogeneit*"[tiab] OR "fibroblast hetero-geneit*"[tiab]**	184,841
#1	**"Fibroblasts"[Mesh] OR "fibroblast*"[tiab] OR "myofibroblast*"[tiab]**	353,023

Embase.com Session Results (28 Sep 2022)

Search	Query	Items found
#5	**#4 NOT ('conference abstract'/it OR 'conference review'/it)**	3,197
#4	**#1 AND #2 AND #3**	4,268
#3	**('cells'/exp AND 'etiology'/lnk) OR 'cell lineage'/exp OR (('cell':ab,ti,kw OR 'cells':ab,ti,kw OR 'cellular':ab,ti,kw OR 'intercellular':ab,ti,kw OR 'peripheral':ab,ti,kw OR 'fibroblast*':ab,ti,kw OR 'bone marrow':ab,ti,kw) AND ('derive*':ab,ti,kw OR 'derivi*':ab,ti,kw OR 'origin*':ab,ti,kw OR 'source':ab,ti,kw OR 'progenitor*':ab,ti,kw OR 'provenance':ab,ti,kw OR 'root':ab,ti,kw OR 'rooted':ab,ti,kw OR 'chimer*':ab,ti,kw**	1,885,088

Search	Query	Items found
	**OR 'germ line'/exp OR 'germ line*':ab,ti,kw OR 'lineage*':ab,ti,kw))**	
#2	**'stem cell transplantation'/de OR 'hematopoietic stem cell transplantation'/exp OR 'bone marrow transplantation'/exp OR (('bone marrow':ab,ti,kw OR 'hematopoetic':ab,ti,kw OR 'hematopoietic':ab,ti,kw) AND ('transplant*':ab,ti,kw OR 'graft*':ab,ti,kw OR 'transfus*':ab,ti,kw OR 'transfer*':ab,ti,kw)) OR 'genetic heterogeneity'/de OR 'cell heterogeneity'/exp OR 'genetic heterogeneit*':ab,ti,kw OR 'genetic hetero-geneit*':ab,ti,kw OR 'fibroblast heterogeneit*':ab,ti,kw OR 'fibroblast hetero-geneit*':ab,ti,kw**	321,780
#1	**'fibroblast'/exp OR 'fibroblast*':ab,ti,kw OR 'myofibroblast*':ab,ti,kw**	354,020

Web of Science (Core Collection) Session Results (28 Sep 2022)

Search	Query	Items found
#4	**#1 AND #2 AND #3**	2,696
#3	**TS=(("cell" OR "cells" OR "cellular" OR "intercellular" OR "peripheral" OR "fibroblast*" OR "bone marrow") AND ("derive*" OR "derivi*" OR "origin*" OR "source" OR "progenitor*" OR "provenance" OR "root" OR "rooted" OR "chimer*" OR "germ line*" OR "lineage*"))**	1,221,108
#2	**TS=((("bone marrow" OR "hematopoetic" OR "hematopoietic") AND ("transplant*" OR "graft*" OR "transfus*" OR "transfer*")) OR "genetic heterogeneit*" OR "genetic hetero-geneit*" OR "fibroblast heterogeneit*" OR "fibroblast hetero-geneit*")**	174,999
#1	**TS=("fibroblast*" OR "myofibroblast*")**	315,514
